# Risk factors for systemic inflammatory response syndrome after endoscopic lithotripsy for upper urinary calculi

**DOI:** 10.1186/s12894-023-01230-9

**Published:** 2023-04-11

**Authors:** Siming Ye, Wei Wang, Zhenliang Yu, Jie Luo

**Affiliations:** 1 Urology Surgery, The First Affiliated Hospital of Zhejiang University School of Medicine, Hangzhou, 310006 China; 2 Nursing Department, The First Affiliated Hospital of Zhejiang University School of Medicine, Hangzhou, 310006 China; 3 Department of Emergency, Shaoxing People’s Hospital, Zhejiang University Shaoxing Hospital), Shaoxing, 312000 China

**Keywords:** Systemic inflammatory response syndrome, Risk factors, Endoscopic lithotripsy, Upper urinary calculi, Retrospective study

## Abstract

**Background:**

To explore the risk factors for systemic inflammatory response syndrome (SIRS) after endoscopic lithotripsy for upper urinary calculi.

**Methods:**

This retrospective study included patients with upper urinary calculi who underwent endoscopic lithotripsy in the First Affiliated Hospital of Zhejiang University between June 2018 and May 2020.

**Results:**

A total of 724 patients with upper urinary calculi were included. One hundred and fifty-three patients developed SIRS after the operation. The occurrence of SIRS was higher after percutaneous nephrolithotomy (PCNL) compared with ureteroscopy (URS) (24.6% vs. 8.6%, P < 0.001) and after flexible ureteroscopy compared with ureteroscopy (fURS) (17.9% vs. 8.6%, P = 0.042). In the univariable analyses, preoperative infection history (P < 0.001), positive preoperative urine culture (P < 0.001), history of kidney operation on the affected side (P = 0.049), staghorn calculi (P < 0.001), stone long diameter (P = 0.015), stone limited to the kidney (P = 0.006), PCNL (P = 0.001), operative time (P = 0.020), and percutaneous nephroscope channel (P = 0.015) were associated with SIRS. The multivariable analysis showed that positive preoperative urine culture [odds ratio (OR) = 2.23, 95% confidence interval (CI): 1.18–4.24, P = 0.014] and operative methods (PCNL vs. URS, OR = 2.59, 95% CI: 1.15–5.82, P = 0.012) were independently associated with SIRS.

**Conclusion:**

Positive preoperative urine culture and PCNL are independent risk factors for SIRS after endoscopic lithotripsy for upper urinary calculi.

## Background

Upper urinary calculus (including renal and ureteral calculi) is a common urologic disease, with a lifetime incidence of 1-20% [1]. The spontaneous passage rates are estimated to be 68% for stones < 5 mm and 47% for stones of 5–10 mm, but spontaneous passage may take more than 1 week [2]. Stones that do not pass spontaneously require interventions, but the paradigm evolved from traditional open surgery to minimally invasive methods [3], such as percutaneous nephrolithotomy (PCNL), ureteroscopy (URS), and flexible ureteroscopy (fURS) [4]. While PCNL involves the creation of a channel between the skin and the kidney, URS uses a rigid ureteroscope through natural ways to reach the stone, while fURS involves a smaller-caliber ureteroscope that carries smaller risks of damaging the ureter [4].

Urosepsis after endoscopic lithotripsy can seriously threaten the patients’ lives; its incidence is 9.8-37% after PCNL and 3.4-8.1% after URS [5]. Systemic inflammatory response syndrome (SIRS) is closely related to urosepsis [1]. Urosepsis is a clinical emergency characterized by the absence of typical early symptoms and rapid progression [6]. There is no specific treatment for SIRS, which can have serious morbidity and mortality [6, 7]. SIRS is the first stage of urosepsis and is often present during the whole course of urosepsis [6].

Identifying risk factors for SIRS and predicting the occurrence of SIRS is essential to screen patients and take preventive measures to avoid patient deterioration. The occurrence of SIRS after fURS is related to hydronephrosis, stone burden, length of operation, preoperative urinary white blood cell (WBC) count, positive preoperative urine culture, diabetes, and other chronic diseases [8]. Liu et al. [9] showed that diabetes, stone burden, longer operation, increased intraoperative irrigation, and infectious stone components were associated with urosepsis after PCNL for upper urinary tract calculi. Chen et al. [10] showed that the number of tracts, blood transfusion, stone long diameter, and pyelocaliectasis were risk factors for SIRS after PCNL.

Although there are many studies on the topic, their results are inconsistent and might differ among hospital levels or populations. Besides, some previous studies only included limited parameters. Determining risk factors from multiple populations is necessary to reach a consensus. In addition, most previous studies focused on only one technique (e.g., PCNL or URS), and only a few examined both PCNL and URS [11]. Furthermore, most studies rely on all data available from the patient charts. Nurses are at the frontline of patient care and are most susceptible to detecting SIRS early. Therefore, this study aimed to explore the independent risk factors for SIRS after endoscopic lithotripsy for upper urinary calculi, based on measurements easily obtainable by nurses, likevital signs,laboratory examination, imaging examination, and surgical records, We hypothesized the result would allow the nurses to make on-the-spot clinical decisions independently or report to physicians in time, to ensure the optimal care of the patients.

## Methods

### Study design and patients

This retrospective study included patients with upper urinary calculi who underwent endoscopic lithotripsy in the urology ward of the First Affiliated Hospital of Zhejiang University between June 2018 and May 2020. SIRS was diagnosed according to the Guidelines for the diagnosis and treatment of urological diseases in China (2014 edition [12]. The inclusion criteria were 1) ≥ 18 years of age, 2) upper urinary calculi diagnosed by imaging examination (including abdominal computed tomography, plain abdominal X-ray, and kidney, ureter, and bladder X-ray), and 3) underwent unilateral or bilateral PCNL, fURS, or URS. The exclusion criteria were (1) SIRS caused by other systemic infections after the operation, (2) history of other operations within 3 months, (3) preoperative basal heart rate ≥ 90 beats/min, (4) immunocompromised patients, (5) malignant tumor, or (6) incomplete clinical data. This study was approved by the Clinical Research Ethics Review Committee of the First Affiliated Hospital, Zhejiang University School of Medicine (#2022 Expedited Review Number 203). The Clinical Research Ethics Review Committee of the First Affiliated Hospital, Zhejiang University School of Medicine, waived the requirement of informed consent because of the retrospective nature of the study.

All patients underwent urine cultures before the operations. They received preoperative antibiotics 1 day before surgery.

### Endoscopic lithotripsy

All patients underwent endoscopic lithotripsy (URS, fURS, or PCNL) routinely.

#### URS

After anesthesia, the patient was set in the bladder lithotomy position. The ureteroscope was placed into the bladder through the urethra and under the action of perfusion water pressure, and the bladder and bilateral ureteral orifice were observed for any abnormality. The zebra guidewire was inserted into the affected ureter as a guide, the perfusion water pressure was adjusted, and the ureteroscope was slowly placed under direct vision to observe whether there were calculi and stenosis throughout the ureter. After the calculi were found, the holmium laser fiber was inserted, the calculi were crushed to less than 2 mm, the fiber was pulled out, and part of the gravel was clamped to the bladder using foreign body forceps or directly pulled out of the body, and a double J tube was indwelled.

#### fURS

In the same way as for conventional URS, the ureteroscope was inserted into the ureter to observe whether there were calculi and stenosis throughout the ureter. Then, the ureteroscope was withdrawn, a soft ureteroscope sheath was placed into the ureteroscope, and a soft ureteroscope was placed into the renal pelvis. The calculi in the renal pelvis were found, and the conditions in the renal calyces were observed. Indwelling double J tube guided by zebra guidewire was performed.

#### PCNL

Under ureteroscopy, a ureteral catheter was inserted into the affected side under the guidance of a zebra guidewire, and water dripping through the catheter was used to form transient artificial hydronephrosis. The patient was turned over, and the incision area was prepared routinely. The needle was inserted into the middle calyces of the affected kidney under B-mode ultrasound guidance, the guidewire was inserted, and the needle was withdrawn through a 1-cm incision of the skin at the puncture site. The fascia dilator was expanded to the appropriate circumference (F8-F24), and the nephroscope channel was established to find the stone. A holmium laser or ultrasonic stone crusher was inserted to break the stone, which was then flushed out. A zebra guidewire was inserted in the direction of the guide wire, and a double J tube was inserted. After careful examination of the visual area confirming no residual stone, the ureteroscope was withdrawn, and a nephrostomy tube with appropriate circumference was inserted.

### Data collection

The patient demographic information and clinical characteristics were retrieved from the hospital’s electronic medical record system. The demographic information included age, sex, admission time, sex, height, weight, and body mass index (BMI). The clinical indicators included imaging and hematological examination results, operative methods (URS, fURS, and PCNL), diabetes history, history of other chronic diseases (cardiovascular and cerebrovascular diseases, hepatic insufficiency, and renal insufficiency), stone location (involving the ureter or limited to the kidney), stone long diameter, degree of hydronephrosis, anatomical abnormality or not, history of operation on the affected side, preoperative infection history, operative methods, operation time, percutaneous nephroscope channel, staghorn calculi or not, preoperative urine culture results, and length of hospital stay, solitary kidney or not, and simultaneous bilateral surgery or not.

### Statistical analysis

Data entry was carried out independently by two researchers in Microsoft Excel;the datasets were compared, and discrepancies were verified and corrected. SPSS 19.0 (IBM, Armonk, NY, USA) was used for statistical analysis. The continuous data conforming to the normal distribution were presented as means ± standard deviation and analyzed using one-way analysis of variance (ANOVA). The categorical data were presented as n (%) and analyzed using the chi-square test. Bonferroni’s method was used for multiple comparisons. Multivariable logistic regression analysis was used to identify the independent risk factors associated with SIRS. Two-sided P-values < 0.05 were considered statistically significant.

## Results

Seven hundred and forty-one patients were included. Two patients with SIRS caused by other systemic infections and 15 patients with incomplete clinical data were excluded. Finally, 724 patients were included (Table 1). Patients who underwent fURS had a higher frequency of operation history on the affected side than those who underwent PCNL (44.4% vs. 29.4%, P = 0.003). The frequency of staghorn calculi was higher in the PCNL group compared with URS and fURS (26.3% vs. 3.2% and 4.6%, both P < 0.001). Stones were larger in the PCNL group compared with URS and fURS (2.5 ± 1.3 vs. 1.2 ± 0.5 vs. 1.6 ± 0.7 cm, all P < 0.001). The frequency of calculi limited to the kidney was higher in the PCNL group, followed by fURS and URS (66.9% vs. 49.0% vs.11.8%, all P < 0.001). There was a significant difference among the PCNL, URS, and fURS groups regarding operation time (66 ± 30 vs. 40 ± 26 vs. 59 ± 29 min, P = 0.002). Finally, the occurrence of SIRS was higher with PCNL compared with URS (24.6% vs. 8.6%, P < 0.001).

**Table 1:** Characteristics of the patients

Item	PCNL (n = 480)	URS (n = 93)	fURS (n = 151)	P
Age (years)	55.7 ± 12.2	55.4±13.7	52.7±13.5	0.083
Sex, n (%)				0.521
Male	357 (74.4)	74 (79.6)	111 (73.5)	
Female	123 (25.6)	19 (20.4)	40 (26.5)	
Body mass index (kg/m^2^)	23.9±3.3	24.0±3.0	24.2±3.8	0.491
Diabetes	69 (14.4)	11 (11.8)	24 (15.9)	0.680
Other chronic diseases^*^, n (%)	66 (13.8)	10 (10.8)	18 (11.9)	0.668
Preoperative infection history, n (%)	150 (31.3)	22 (23.7)	48 (31.8)	0.276
Positive preoperative urine culture, n (%)	66 (14.2)	11 (11.8)	23 (15.2)	0.755
History of operation on affected side stones, n (%)	141 (29.4)	33 (35.5)	67 (44.4)^a^	0.003
Staghorn calculi, n (%)	126 (26.3)	3 (3.2)^a^	7 (4.6)^a^	< 0.001
Stone long diameter (cm)	2.5±1.3	1.2±0.5^a^	1.6±0.7^ab^	< 0.001
Stone location, n (%)				< 0.001
Involving the ureter	159 (33.1)	82 (88.2)	77 (51.0)	
Limited to the kidney	321 (66.9)	11 (11.8)	74 (49.0)	
Bilateral stones, n (%)	206 (42.9)	42 (45.2)	66 (43.7)	0.898
Degree of hydronephrosis, n (%)				0.062
Severe	140 (29.2)	29 (31.2)	31 (20.5)	
Non-severe	340 (70.8)	64 (68.8)	120 (79.5)	
Anatomical abnormality, n (%)				0.051
Abnormal	68 (14.2)	24 (25.8)	26 (17.2)	
Normal	412 (85.8)	69 (74.2)	125 (82.8)	
Solitary kidney, n (%)	1 (0.2)	3 (3.2)	6 (4.0)	0.001
Simultaneous bilateral surgery, n (%)	17 (3.5)	4 (4.3)	6 (4.0)	0.925
Operation time (min)	66±30	40±26	59±29	0.002
Percutaneous nephroscope channel	20.4±3.5	0	0	---
SIRS	118 (24.6)	8 (8.6)^a^	27 (17.9)	0.001

PCNL: percutaneous nephrolithotomy; URS: ureteroscopy; fURS: flexible ureteroscopy; SIRS: systemic inflammatory response syndrome

^a^: P < 0.017 compared to PCNL group; ^b^: P < 0.017 compared to URS group

The univariable and multivariable logistic regression analyses are shown in Table 2. In the univariable analyses, preoperative infection (OR = 2.365, 95% CI: 1.597–3.503, P < 0.001), positive preoperative urine culture (OR = 3.018, 95% CI: 1.844–4.940, P < 0.001), history of kidney operation on the affected side (OR = 1.478, 95% CI: 1.001–2.183, P = 0.049), staghorn calculi (OR = 2.991, 95% CI: 1.934–4.626, P < 0.001), stone long diameter (OR = 1.184, 95% CI: 1.033–1.357, P = 0.015), stone limited to the kidney (OR = 0.595, 95% CI: 0.410–0.863, P = 0.006), operative methods (PCNL vs. URS, OR = 1.174, 95% CI: 1.073–3.490, P = 0.001), operative time (OR = 1.009, 95% CI: 1.003–1.014, P = 0.020) were associated with SIRS. Percutaneous nephroscope channel (OR = 1.079, 95% CI: 1.015–1.147, P = 0.015) was associated with SIRS in patients who underwent PCNL. The multivariable analysis showed that positive preoperative urine culture (OR = 2.24, 95% CI: 1.17–4.22, P = 0.014) and operative methods (PCNL vs. URS, OR = 2.59, 95% CI: 1.15–5.82, P = 0.021) were independent risk factors for SIRS.

**Table 2:** Univariable and multivariable analysis of postoperative SIRS

Variables	Univariable analysis		Multivariable analysis	
	OR (95%CI)	P	OR (95%CI)	P
Age	0.991 (0.977, 1.005)	0.213		
Sex	0.698 (0.471, 1.036)	0.073		
Body mass index	0.994 (0.968, 1.022)	0.676		
Diabetes	1,217 (0.719, 2.062)	0.464		
Other chronic diseases				
No	Ref			
Yes	0.879 (0.493, 1.565)	0.660		
Preoperative infection history	2.365 (1.597, 3.503)	< 0.001	0.867 (0.513, 1.465)	0.593
Positive preoperative urine culture	3.018 (1.844, 4.940)	< 0.001	2.224 (1.172, 4.220)	0.014
History of operation on affected side stones	1.478 (1.001, 2.183)	0.049	0.905 (0.607, 1.351)	0.627
Staghorn calculi	2.991 (1.934, 4.626)	< 0.001	1.624 (0.987, 2.696)	0.061
Stone long diameter	1.184 (1.033, 1.357)	0.015	0.914 (0.758, 1.101)	0.343
Stone location				
Limited to the kidney	0.595 (0.410, 0.863)	0.006	1.287 (0.845, 1.960)	0.240
Involving the ureter	Ref		Ref	
Bilateral stones				
No	Ref			
Yes	0.995 (0.694, 1.427)	0.979		
Degree of hydronephrosis				
Severe	0.909 (0.607, 1.362)	0.645		
Non-severe	Ref			
Anatomical abnormality	1.267 (0.797, 2.013)	0.371		
Solitary kidney	0.411 (0.052, 3.268)	0.385		
Operative methods				
Percutaneous nephrolithotomy	1.174 (1.073,3.490)	0.001	2.590(1.152,5.820)	0.021
Flexible ureteroscopy	1.097 (0.988,1.219)	0.083	1.893(0.799,4.485)	0.147
Ureteroscopy	Ref			
Simultaneous bilateral surgery, n (%)				
No	Ref			
Yes	0.834 (0.314, 2.264)	0.735		
Operation time	1.009 (1.003, 1.014)	0.020	1.006 (1.000, 1.012)	0.065
Percutaneous nephroscope channel	1.079 (1.015, 1.147)	0.015		

## Discussion

The results suggest that positive preoperative urine culture and nephrolithotomy are independent risk factors for SIRS after endoscopic lithotripsy for upper urinary calculi. The results might help patient management and prevent the early deterioration of patients with SIRS by reducing the nursing risk.

In this study, the occurrence of SIRS after lithotripsy was 21.1%, comparable to the rates reported in previous studies (11% [8] and 23% [10]). In the univariable analysis, SIRS was associated with preoperative infection, positive preoperative urine culture, history of kidney operation on the affected side, staghorn calculi, stone long diameter, limited to the kidney, PCNL, operative time, and percutaneous nephroscope channel. Those results are supported by previous studies [8–10]. The present study included patients who underwent PCNL or URS, and the results showed that PCNL was an independent risk factor for SIRS compared with URS. Indeed, PCNL involves creating a channel from the skin to the kidney, while URS uses natural channels, avoiding surgical trauma. fURS was not associated with SIRS compared with URS as both use natural channels. URS is associated with a risk of SIRS [5, 13]. Chung et al. [14] showed that the complication rates were lower for PCNL than for URS. Xia et al. [15] showed that PCNL was a better choice than URS to reduce sepsis, especially in patients with positive urine cultures, but the risk factors for sepsis might differ from those of SIRS.

Preoperative positive urine culture is generally associated with or a risk factor for SIRS and urosepsis [8, 16]. The presence of pathogens in urine (usually a sterile environment) is bound to induce inflammation and increase the risk of inflammatory reactions.

Still, the present study reported a positive association between the percutaneous nephroscope channel and SIRS in patients who underwent PCNL, while many other studies reported a negative association, as reviewed by Bhojani et al. [17]. The operation time was not independently associated with SRIS in the present study, which conflicts with a previous study [8], possibly because the operation time was significantly different among patients who received URS, fURS, and PCNL. In addition, the literature generally supports that diabetes, female sex, and obesity are associated with SIRS [17], but the present study did not support these associations. Nevertheless, various studies reported different factors associated with SIRS [8–10]. The discrepancies can be due to the choice of variables to be analyzed, the study populations, and local practices. The author’s center is a first-class tertiary hospital with a department specializing in calculus management. The preoperative examinations are careful, and attention is paid to perioperative blood glucose control. For patients with diabetes, the appropriate time for blood glucose measurement is fasting and 2 h after meals. The perioperative blood glucose levels are controlled at ≤10.0 mmol/l for fasting blood glucose and ≤16.7 mmol/l for postprandial blood glucose, avoiding the risk of infection caused by hyperglycemia [18]. For patients with severe renal insufficiency, the center adopts fistulation improvement before elective surgery, which helps prevent SIRS. Moreover, 10 patients had solitary kidneys, and 27 underwent simultaneous bilateral surgery, which was insufficient for subgroup analyses. Furthermore, preoperative urinary tract infection control failed due to the restriction of antibiotics. Gram-negative bacteria are the most common bacteria in urine cultures at the authors’ center, and the drug resistance rate is high. The effect of prevention and treatment of infection is limited due to the resistance to common antibiotics. Furthermore, the stones release bacteria and toxins after being crushed by the laser. Due to the intraoperative high-pressure continuous perfusion, the small renal vessels and lymphatic spaces are opened, resulting in many bacteria and toxins entering the systemic circulation. In PCNL, the risk of infection increases due to the open, percutaneous nephroscope channel during nephrolithotomy. Therefore, for patients after PCNL, nurses should pay attention to classifying and paying close attention to vital signs and infection indexes.

This study had strengths. First, it analyzed PCNL, URS, and fURS within the same study, which has been done only once [11]. Furthermore, most studies rely on all data available from the patient charts, while this study was nurse-oriented, which is clinically relevant since nurses are at the frontline of patient care. Still, this study had several limitations. The first limitation of the study is its retrospective nature and the inherited bias. Besides, only a single center was included, and the sample size was small. In addition, the local practices might have influenced the identification of the risk factors. Finally, because it was a retrospective study, only the variables found in the patient charts could be analyzed.

The medical system in China is still highly oriented “from top to bottom”, meaning that physicians usually make almost all clinical decisions. In this study, the variables are easy to obtain by nurses, allowing them to make on-the-spot clinical decisions independently or reported to physicians in time, to ensure the optimal care of the patients. Indeed, identifying patients at higher risk of SIRS and predicting the occurrence of SIRS before it occurs is essential to screen patients and take preventive measures to avoid patient deterioration. Since SIRS can occur and deteriorate rapidly, it would allow the nurses to detect it early and take early measures pending the physician visit.

## Conclusions

In conclusion, positive preoperative urine culture and PCNL might increase the risk of SIRS after endoscopic lithotripsy for upper urinary calculi. Those factors are readily available in the medical charts and should prompt nurses to increase their vigilance toward developing SIRS.

## Data Availability

All data generated or analyzed during this study are included in this published article.

## List of abbreviations

SIRS: systemic inflammatory response syndrome
PCNL: percutaneous nephrolithotomy
CI: confidence interval
WBC: white blood cell
BMI: body mass index
URS: ureteroscopy
fURS: flexible ureteroscopy
